# A Brief Review of Bone Cell Function and Importance

**DOI:** 10.3390/cells12212576

**Published:** 2023-11-05

**Authors:** Veronika Šromová, Dinara Sobola, Pavel Kaspar

**Affiliations:** 1 Department of Biomedical Engineering, Faculty of Electrical Engineering and Communication, Brno University of Technology, 601 90 Brno, Czech Republic; 2 Department of Physics, Faculty of Electrical Engineering and Communication, Brno University of Technology, 601 90 Brno, Czech Republic; kasparp@vut.cz; 3 Academy of Sciences of the Czech Republic, Institute of Physics of Materials, Žižkova 22, 616 62 Brno, Czech Republic

**Keywords:** bone cells, physiology of bone tissue, microscopic and macroscopic intricacies

## Abstract

This review focuses on understanding the macroscopic and microscopic characteristics of bone tissue and reviews current knowledge of its physiology. It explores how these features intricately collaborate to maintain the balance between osteoblast-mediated bone formation and osteoclast-mediated bone resorption, which plays a pivotal role in shaping not only our physical framework but also overall health. In this work, a comprehensive exploration of microscopic and macroscopic features of bone tissue is presented.

## 1. Introduction

The human skeletal system is a marvel of biological engineering. From the explanation of the main functions of bones and their composition to the precise mechanisms of bone formation and remodeling, we seek to unravel and shed light on the pivotal roles that bones play beyond mere structural support with a focus on research and insights from the fields of physiology, anatomy, and histology of bone tissue.

Bones are not just lifeless scaffolds; they are living, dynamic organs that play crucial roles in support, protection, movement, and even blood cell production. Bone distinguishes itself from other connective tissues through its rigidity and firmness. These distinctive qualities arise from the incorporation of inorganic salts into a matrix composed of collagen fibers, non-collagenous proteins, and so on. Furthermore, bone possesses the remarkable ability to self-repair, adapting its shape, mass, and other properties in response to mechanical demands.

## 2. Overview of Bone Physiology and Structure

### 2.1. Main Functions of Bone Tissue

The main functions of bone tissue include:

- Structural support;
- Protection of internal organs and soft tissues from damage;
- Locomotion;
- Mineral storage;
- Production of blood cells;
- Endocrine regulation.

The basic mechanical property of bone tissue is its strength, which provides both support and protection and ensures a framework for the body [1]. Skeletal muscles attach to bones via tendons, and when a skeletal muscle contracts, it produces a pulling force that is transmitted through the tendon to the bone. This interaction between the muscle and the bone is what enables the movement of the body [2].

Bone tissue also plays an important role as a mineral store, especially for calcium and phosphorus. These minerals are involved in various metabolic processes within our body and can be released into the bloodstream when needed. In situations where the calcium level in the blood is low, specialized cells in the bone, known as osteoclasts, break down bone tissue, releasing the minerals into the bloodstream. Conversely, osteoblasts, another type of bone cell, deposit calcium and phosphate into bone tissue when blood calcium levels are high, reducing the amount of calcium circulating in the bloodstream. This intricate process is highly regulated by various hormones and signaling pathways (one of the most important hormones involved in this regulatory system is the parathyroid hormone PTH). In summary, the mineral storage function of bone tissue is essential for maintaining the balance of minerals in the body and ensuring optimal physiological function [3,4].

Bones may appear inert and rock-solid, but in reality, they are porous inside and house the bone marrow, which is responsible for the production of blood cells. The process of producing mature blood cells from precursor cells is called hematopoiesis or hematopoiesis. Bone marrow contains stem cells such as pluripotent and hematopoietic stem cells, which can proliferate and differentiate to give rise to different types of blood cells, including red blood cells (erythrocytes), white blood cells (leucocytes), and platelets (thrombocytes) [5].

The function of an endocrine organ is to control remote functions by releasing either a peptide or a steroid hormone. Bone cells can function as endocrine cells and regulators of many metabolic processes by secreting specific hormones and signaling molecules [6]. For example, fibroblast growth factor 23 (FGF-23) is produced and released by osteocytes and osteoblasts. FGF-23 is a hormone involved in bone remodeling, phosphate metabolism, and vitamin D regulation. It acts primarily by decreasing the expression of sodium-phosphate cotransporters in the proximal tubule of the kidney, thereby inhibiting phosphate reabsorption [7]. Furthermore, bone cells secrete at least two more hormones, sclerostin and osteocalcin. Some studies show that osteocalcin, primarily produced and secreted by osteoblasts, plays a significant role in the regulation of energy metabolism, glucose tolerance, testosterone production, and bone resorption [6,8]. Other studies have indicated that osteocytes are capable of producing a protein called sclerostin, which acts in a paracrine manner and impedes the differentiation of osteoblasts [8].

### 2.2. Microstructure of Bone Tissue

**Figure 1:**
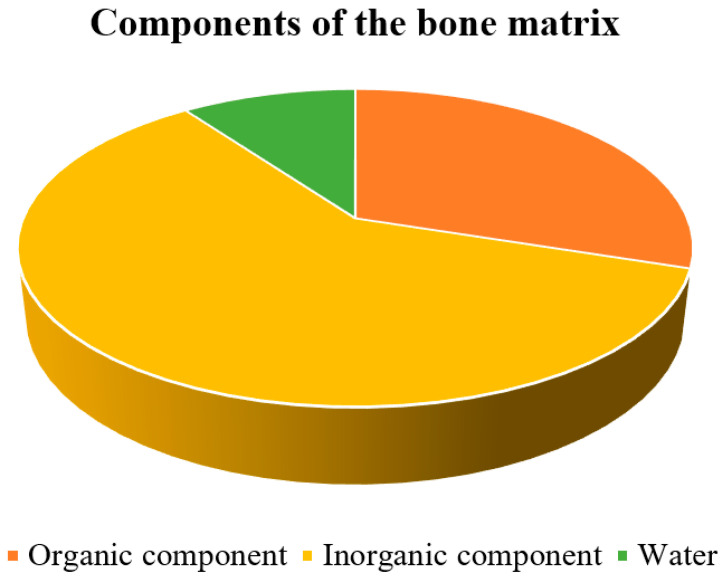
Distribution of bone matrix components.

Bone is a mineralized tissue consisting of about 60% inorganic components, mainly hydroxyapatite, along with 10% water and 30% organic components (Figure 1), primarily proteins [9]. Because of its combination of inorganic and organic elements, it is one of the most rigid structures in the body. The organic element of the bone matrix is primarily collagen, a fibrous protein that gives bone flexibility and tensile strength [10]. Collagen makes up a large portion of the organic matrix, accounting for approximately 85 to 90% of the organic matrix of bone [11].

#### 2.2.1. Organic Component of the Bone Matrix

Collagen fibers are found in all types of connective tissue and are made up of the protein collagen. These fibers are very tensile (an elongation of 10 to 20% may result in a fiber break) and flexible and can form either sparse networks of thin collagen fibrils or dense bundles depending on the function and placement [12].

Among the different types of collagens, type I collagen is the predominant form found in the bone matrix. It forms the characteristic triple helix structure, provides structural support, and contributes to the mechanical properties of bone, such as its flexibility and resistance to tensile forces. Type III and type V collagen occur in trace amounts, where type III collagen provides additional flexibility to the bone tissue, and type V collagen helps regulate the structure and organization of collagen fibers in the bone matrix. FACIT (Fibril-Associated Collagens with Interrupted Triple Helices) collages are also present in the bone matrix and have a very similar function to type V collagen [11].

Non-collagenous proteins make up approximately 10 to 15% of the total protein content in bone. These proteins play important roles in various biological processes, including mineralization, bone remodeling, cell signaling, and regulation of bone cell activity [11].

These non-collagenous proteins include, for example [6,8,13]:

- Osteocalcin—regulates bone formation and the activity of osteoclasts and their precursors; osteocalcin also plays a pivotal role in the hormonal regulation of insulin metabolism and as a mediator of testosterone secretion.
- Osteonectin—can modulate the activity of growth factors, influences cell adhesion, and plays a role in the mineralization and deposition of hydroxyapatite.
- Osteopontin—regulates bone formation, migration, adhesion, and mineralization processes.
- Bone sialoprotein—binds Ca^2+^ and is involved in mineralization.
- RANKL (Receptor Activator of Nuclear Factor κ-B Ligand)—functions in the regulation of bone resorption and differentiation of osteoclasts from their precursors.

#### 2.2.2. Inorganic Component of the Bone Matrix

The inorganic component of bone primarily consists of minerals, with calcium and phosphate being the most important minerals. These minerals form hydroxyapatite crystals (Ca_10_(PO_4_)_6_(OH)_2_), which are embedded in the collagen matrix. Calcium and phosphate provide hardness and much of the rigidity, providing structural strength, and they contribute to approximately 60–70% of the dry weight of bone [9]. Hydroxyapatite, as a vital component of bone composition, plays an important role not only in maintaining bone structure but also in facilitating bone regeneration, especially in these two key processes: osteoinduction (refers to the mechanism responsible for the generation of new bone tissue and the conversion of immature cells into preosteoblasts, crucial cellular units involved in the development of new bone) and osteoconduction (refers to the ability of bone-forming cells to migrate across a bone structure and replace it with new bone tissue) [14,15].

However, other ions such as sodium, potassium, magnesium, carbonate, barium, and zinc are also present [16]. These ions can reduce the crystallinity of bone minerals, potentially altering specific mineral properties such as solubility (which is significant for maintaining mineral homeostasis and facilitating bone adaptation) [9].

Water also plays a significant role in the composition of bone tissue as it is present within the collagen matrix and helps maintain the hydration and flexibility of the bone. The surface ions of hydroxyapatite are hydrated, and a layer of water and ions forms around the crystals. This hydration layer facilitates ion exchange between the crystals and body fluids [12].

#### 2.2.3. Cells of Bone

In the bone tissue, we distinguish four types of bone cells:

- Osteoclasts;
- Osteoblasts;
- Osteocytes;
- Lining cells.

The first three bone cell types represent different stages in the maturation process of a single cell type. Together with their progenitor cells, they form the osteoblastic cell lineage (a subgroup of the lineage that includes connective tissue cells), while osteoclasts belong to the hematopoietic cell family together with all types of blood cells [13].

Osteoclasts are large cells that have multiple nuclei, which are essential for their high metabolic activity and efficient resorption (breakdown) of bone tissue. The cytoplasm of the osteoclasts contains several well-developed organelles such as mitochondria, endoplasmic reticulum, Golgi apparatus, transport secretory vesicles, and microtubule arrays. One prominent feature of the osteoclast cytoplasm is the presence of a specialized structure called the ruffled border, which is a highly folded cell membrane that forms numerous microvilli-like projections forcing the bone surface. It is the site where bone resorption occurs. Tartrate-resistant acid phosphatase (TRAP) is indeed a lysosomal enzyme that serves as a widely used histological marker for identifying osteoclasts in various tissue sections during histological analysis. It is an enzyme that is involved in the process of bone resorption, where it plays a role in breaking down bone tissue [5,13].

**Figure 2:**
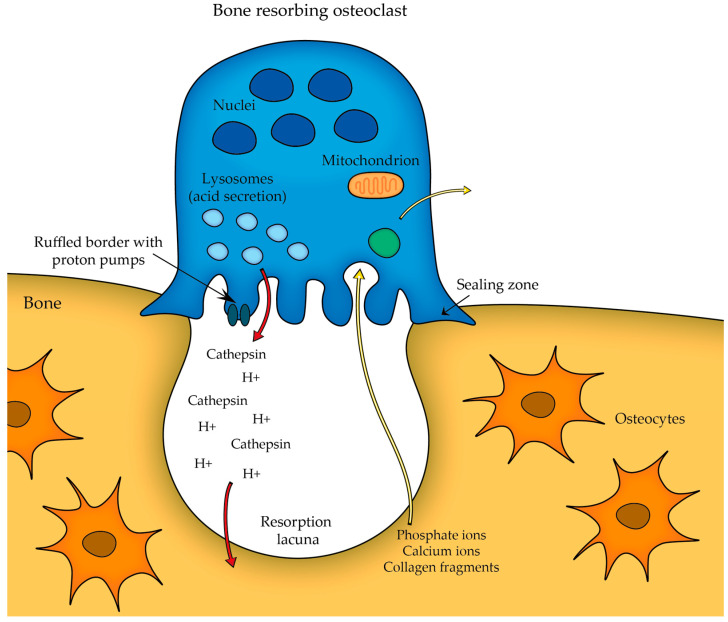
Osteoclast sealing zone formation and proton transport.

Osteoclasts play a pivotal role in bone remodeling. A fully mature osteoclast must maintain a strong attachment to the bone to effectively resorb it—thus, the initial step in osteoclast differentiation involves the attachment of osteoclast precursor to the bone surface. This leads to the formation of a structure known as the sealing zone and the development of the ruffled border membrane rich in proton pumps, which actively transport protons into the resorption lacuna (Figure 2). The acidification of the microenvironment is important in dissolving the inorganic component of the bone matrix. Transcytosis facilitates the removal of degraded products from the resorption lacuna. During this process, the degraded products are first endocytosed at the ruffled membrane; then, they are transported along a transcytotic vesicular pathway within the cell before being released at the anti-resorptive side of the cell [9,13].

The attachment mechanism of osteoclasts to the bone surface allows these cells to concentrate their resorptive activity in a specific area. Integrins (cell adhesion molecules expressed by osteoclasts) are responsible for binding to specific extracellular matrix proteins in bone, such as collagen (the integrin α_2_β_1_) and other bone matrix proteins (integrin α_V_β_3_ binds to osteopontin, bone sialoprotein, etc.). Furthermore, osteoclasts have a unique actin-ring structure localized near the adhesion receptors. This specific structure ensures an efficient and localized resorption of bone [13].

While the osteoclast attaches to the bone surface, the actual bone resorption takes place exactly at the ruffled border (as seen in Figure 2), which serves as the site where the osteoclast secretes acid (protons H^+^) that acidifies the local environment and other lysosomal enzymes (e.g., cathepsin K, which is responsible for degrading the organic components of the bone matrix) to dissolve the mineralized bone matrix. The acidic conditions help to dissolve the hydroxyapatite crystals present in the bone, releasing calcium and phosphate ions into the resorption cavity. Thus, the combination of acidic conditions and lysosomal enzymes effectively degrades the bone matrix [13].

**Figure 3:**
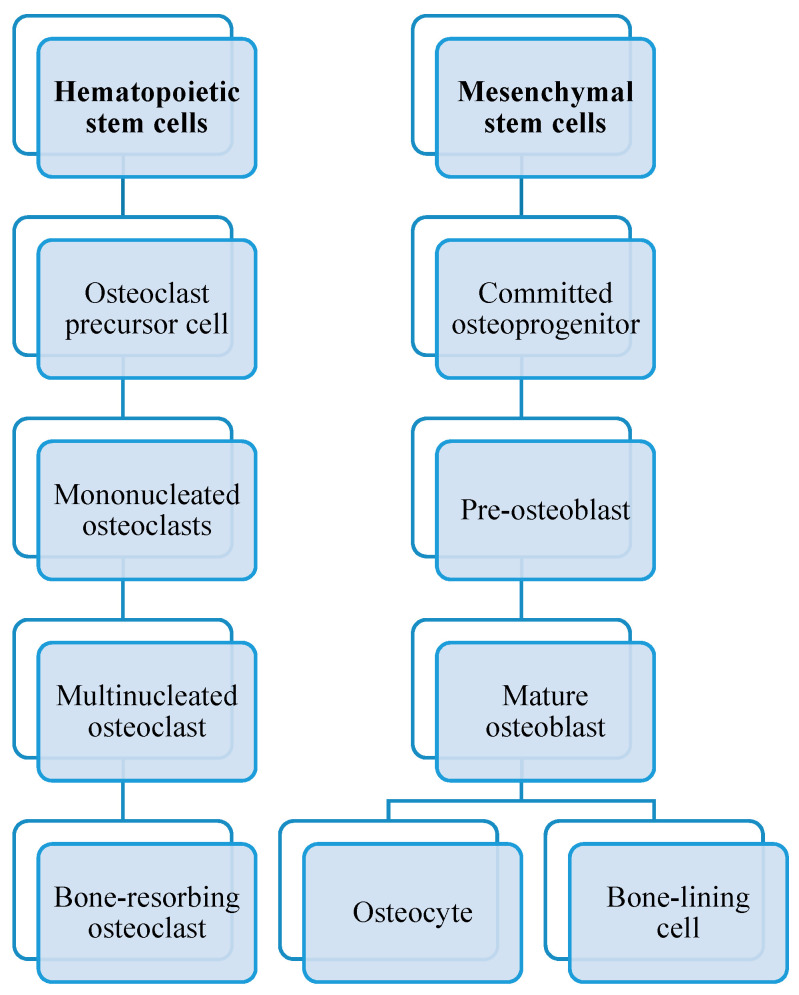
Differences in osteoblastogenesis and osteoclastogenesis.

As you can see in Figure 3, osteoclasts originate from hematopoietic stem cells. They belong to the monocyte/macrophage family. Initially, hematopoietic stem cells develop into macrophage colony-forming units CFU-M when exposed to the M-CSF precursor. After activation by the signal RANKL-RANK, the major osteoclastogenic protein that is able to recognize its receptor RANK on bone marrow macrophages, the macrophage colony-forming units differentiate into mononuclear osteoclasts that assemble into multinucleated osteoclasts. The final stages of osteoclast differentiation must interact directly with cells from the bone marrow stroma or those belonging to the osteoblast lineage to be fully mature [17].

The role of bone morphogenetic proteins (BPMs) is to stimulate or induce mesenchymal stem cells to differentiate into osteoblasts. These proteins play essential roles in various biological processes (particularly in tissue regeneration) and belong to the transforming growth factor-beta (TGF-β) superfamily of proteins. Hence, osteoblasts arise from multipotent mesenchymal precursors, and when BPMs bind to their specific receptors, these signals activate specific genes that promote the expression of transcription factors, such as Runt-related transcription factors 2, RUNX2, and Osterix, which are crucial for osteoblast formation. Mesenchymal stem cells undergo a series of changes, leading to their differentiation into pre-osteoblasts and then mature osteoblasts, which are responsible for the synthesis and secretion of bone matrix proteins, etc. Mature osteoblasts can undergo three different fates depending on the needs of the bone tissue and the surrounding microenvironment: osteocyte differentiation (some mature osteoblasts can transform into osteocytes); apoptosis (programmed cell death; this fate is essential for maintaining the balance between bone formation and resorption); and some mature osteoblasts can become bone-lining cells, which form a thin layer on the surface of the bone and play a role in bone remodeling [13,16,18].

Osteoblasts, in contrast to osteoclasts, are bone-forming cells responsible for synthesizing and storing new bone tissue, as well as regulating osteoclasts. They play a key role in bone formation and remodeling throughout life. Bone remodeling is a continuous and dynamic process in which old or damaged bone tissue is removed by osteoclasts or the bone-resorbing cells (bone resorption is considered to be much faster than bone formation) and replaced by new bone tissue formed by osteoblasts. This process of bone formation and bone resorption must be tightly regulated to maintain bone homeostasis and ensure that bone mass and mechanical strength remain relatively stable over time. In case of disruption or dysregulation of this process, abnormal bone remodeling may occur. Examples of dysregulation include, for example, osteoporosis, osteopetrosis, or rickets (discussed in more detail in the following chapters) [13,18,19,20].

Osteoblasts are mononuclear cells. The cytoplasm contains various cellular organelles such as the enlarged Golgi apparatus (involved in the processing and packaging of bone matrix proteins, such as collagen and osteocalcin, which bind to the hydroxyapatite crystals present in bone, and a number of glycoproteins, before they are secreted into the extracellular space), prominent rough endoplasmic reticulum involved in protein synthesis, many secretory vesicles, etc. [5,13,18,20].

**Figure 4:**
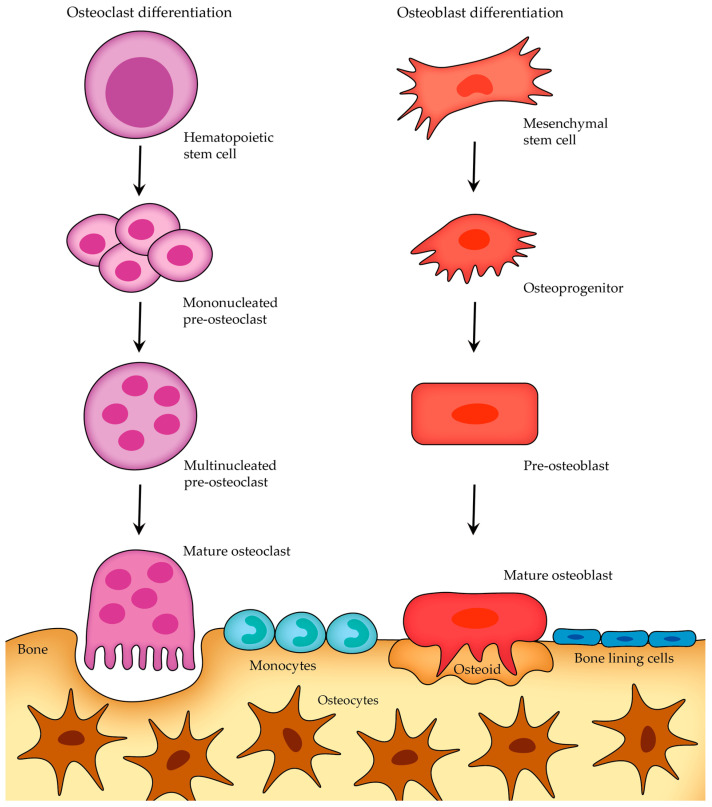
Osteoblast and osteoclast differentiation and life cycle before apoptosis.

Although osteoblasts and osteoclasts have distinct functions and arise from different developmental origins, both cell types undergo a similar short life cycle involving stages of activation, activity, and eventual removal (once they have completed their task, they undergo apoptosis—programmed cell death) (Figure 4). Osteoblasts arise from mesenchymal stem cells, while osteoclasts are derived from hematopoietic stem cells. Once osteoblasts and osteoclasts reach their mature stage, they do not typically migrate very far from their localized environment in contrast to their progenitor cells, which have the ability to travel from distant sites in response to specific signals or stimuli [13].

Bone formation is a two-step process involving matrix formation (osteoblasts produce and deposit unmineralized osteoid) and mineralization (the deposited matrix is subsequently mineralized, i.e., calcium and phosphate ions are incorporated, leading to the formation of hydroxyapatite crystals and hardening of bone tissue). As mentioned earlier, osteoblasts are responsible for the production and release of the unmineralized bone matrix, or ground substance of bone, which is composed of about 90% collagen and about 10% non-collagenous proteins. In other words, almost all bone matrix molecules contain a protein component, and the information synthesis of all proteins, including those present in the bone matrix, is encoded within an organism’s genome. Thus, cellular control over the composition and organization of the bone matrix is achieved by regulating gene expression. Osteoblasts actively transcribe and translate genes that encode bone matrix proteins, resulting in the synthesis and secretion of these proteins into the extracellular space. Once synthesized, proteins may undergo various post-translational modifications, such as hydroxylation, glycosylation, and phosphorylation, which may further influence their functions and interactions within the bone matrix [13,18,20].

Osteoblasts secrete an unmineralized organic matrix called osteoid. This osteoid consists mainly of collagen fibers and other non-collagenous proteins that provide the framework for bone mineralization. As osteoblasts continue their activity, the bone formation front moves away from them, and the osteoid gradually starts to undergo mineralization. This process of mineralization involves the deposition of hydroxyapatite crystals, which transforms the soft osteoid into a rigid and calcified bone matrix [21,22].

**Figure 5:**
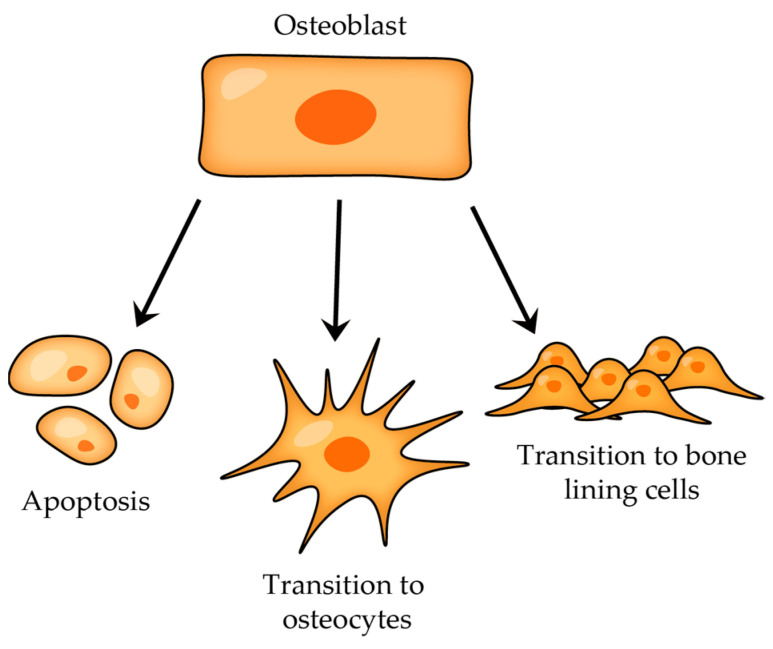
Osteoblast transitions after bone-forming phase.

After the bone-forming phase, osteoblasts can have three different fates (Figure 5): some osteoblasts become osteocytes, which are embedded in the mineralized bone matrix, other osteoblasts can transform into bone-lining cells that cover the bone surface, and a certain subgroup of osteoblasts undergo programmed cell death (apoptosis) [23].

Osteocytes are the most abundant cells in mature adult bone tissue, comprising about 90% to 95% of the cellular component. They originate from osteoblasts (once osteoblasts complete their bone-forming role, some of them become embedded within a lacuna as osteocytes; those that do not experience this transition either become bone-lining cells or undergo apoptosis; Figure 5) and have a distinct morphology characterized by dendritic processes that extend from the cell body, connecting with other bone cells (either osteocytes themselves or with cells on the bone surface) or blood vessels. These processes, along with the canaliculi (tiny channels within the bone), are the primary means of communication for osteocytes with the outside world, and they facilitate the transportation of nutrients and waste products [21,22].

As already mentioned, osteocytes are derived from osteoblasts during the process of bone formation, and that is why there should be identifiable transitional cell stages between them. These intermediate transitional stages can be recognized based on both morphological and molecular properties. In contrast to the cuboidal shape of osteoblasts, osteocytes have a dendritic structure with a rounded cell body, a large nucleus, and cell processes extending from the cell body, which enable the osteocytes to establish connections with neighboring cells both within the bone matrix and on its surface. By observing changes in cell shape and structure, as well as examining specific molecular markers associated with osteocyte differentiation, we can distinguish these three stages in the transition from osteoblast to mature osteocyte [22,23,24]:

- *Type I Pre-osteocyte (Osteoblastic Osteocyte)*—it is not easy to distinguish these pre-osteocytes from osteoblasts due to their similar plump shape and the abundance of cytoplasmic organelles; also, these cells have moved away from the osteoblast layer, yet they remain in direct contact with this layer and connect to the osteoblasts via numerous cell–cell connections [22,23,25],
- *Type II Pre-osteocyte (Osteoid-Osteocyte)*—a type II pre-osteocyte is also called a osteoid-osteocyte, which refers to a cell positioned within the osteoid seam, and furthermore, the ratio of nucleus size to cytoplasmic content is increased, cytoplasmic processes are elongated, and they continue to exhibit a bone-forming role [26],
- *Type III Pre-osteocyte*—type III pre-osteocytes are characterized by being completely separated from the osteogenic lamina, and they appear to lose their bone-secreting activity, which were distinctive traits of the earlier stages; in contrast to type II pre-osteocytes, the type II pre-osteocyte has a smaller cell body with a clearly defined ellipsoid shape, the ratio of nucleus size to cytoplasmic volume is higher, and the content of cytoplasmic organelles is reduced [26]

Now that we have described the various stages of the transformation of the osteoblast into an osteocyte, it is time to describe why and on what basis this transformation occurs.

Over the course of bone formation, the osteocyte processes, dendritic cytoplasmic formations located on the vascular surface of osteocytes (facing the bone surface), continue to extend, allowing osteocytes to maintain contact with the active layer of osteoblasts and regulate their activity. Once these extensions stop growing, they emit a signal that triggers the commitment of an osteoblast to undergo transformation into an osteocyte. Simultaneously, a pre-osteoblast located in close proximity to this committed osteoblast initiates its transformation into a mature osteoblast. This new osteoblast will eventually assume the position within the osteoblast layer that was previously occupied by the committed osteoblast. The cessation of growth in a vascular extension could be triggered by the osteoblasts it was in contact with or could be a consequence of the gradual decrease in the blood supply to the osteocytes as new layers of bone are added [22,23].

The differentiation of osteoblasts is governed by various transcription factors and signaling proteins, which encompass Runx2 (Runt-related transcription factor 2); Osterix (also known as SP7 or Osx); and the Wnt signaling pathway, or Sclerostin. The presence of Runx2 is vital for the determination of the osteoblast lineage and bone development, as evidenced by the absence of both endochondral and membranous bones in Runx2-deficient mice. Osterix is probably downstream of Runx2 (as evidenced by the absence of detectable Osx transcripts in mice that lack functional Runx2 genes) and is expressed in all osteoblasts. It is important for the differentiation of pre-osteoblasts into mature osteoblasts. In other words, Runx2 is the first transcription factor that initiates the commitment of mesenchymal stem cells to the osteoblast lineage (it is noteworthy that Runx2 inhibits the complete maturation of osteoblasts and the subsequent formation of mature bone), while Osterix further promotes the transition of pre-osteoblasts into mature osteoblasts. In embryos lacking Osx, there is a complete absence of bone formation. Sclerostin is considered to be an extracellular Wnt antagonist that interacts with the LRP5/6 receptor. This interaction prevents Wnt binding to LRP5/6, which leads to the inhibition of Wnt signaling [27,28,29].

**Figure 6:**
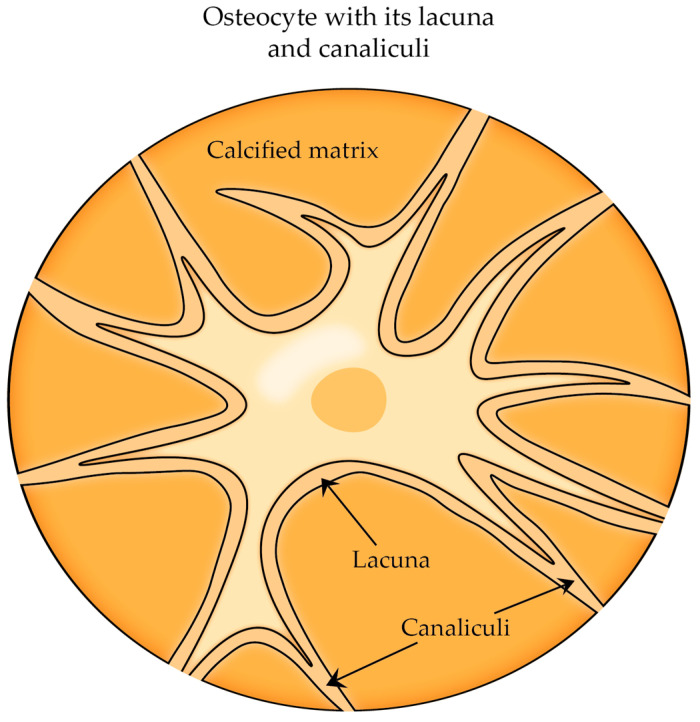
The cell body of osteocyte located in lacuna, and the cytoplasmic processes are situated in canaliculi.

At the end of the osteoblast-to-osteocyte differentiation process, fully developed osteocytes are enclosed within a network of microscopic bony cavities referred to as lacunae (where the cellular bodies are situated) and canaliculi (where the arborization of cytoplasmic processes is contained) and are surrounded by bone fluid responsible for oxygen and nutrient supply (Figure 6) [25,30]. In addition, the bone fluid allows osteocytes to come into contact with hormones and signals from the bloodstream that come from distant areas of the body, like the kidneys and parathyroid glands. Osteocytes extend their processes toward bone surfaces and connect with other osteocytes, osteoblasts, and bone-lining cells. This network also encompasses interactions with osteoclasts, bone marrow cells, and blood vessels [30].

This transformation from osteoblast to osteocyte is an active process. A pivotal factor in this differentiation process is the protein E11, which serves as the initial specific marker expressed by osteocytes [30,31].

Even though osteocytes are relatively abundant within bone tissue, a definitive function for them has not yet been clearly defined. It is believed that osteocytes can function as mechanosensors and form a sensory network responsible for sensing mechanical loads and tissue damage. Furthermore, osteocytes can facilitate communication between deep sites within the bone and the external environment and might serve as agents for repairing the bone matrix located deep within the bone, away from the remodeling activities of osteoblasts and osteoclasts. They are found to be involved in the control of mineral metabolism and in releasing calcium stores within the skeleton, a process particularly notable during lactation. During the second half of the last century, various observations hinted at the possibility of osteocytes engaging in bone resorption and thus enlarging their lacunae. This phenomenon came to be termed “osteocytic osteolysis”, and studies suggested that this process could be prompted by diverse stimuli like parathyroid hormone PTH, restricted calcium intake, hibernation, or reproductive cycles [13,22,25,32].

Osteocytic osteolysis: Even though the concept of osteocytic osteolysis lost favor by the late 1970s as it became evident that osteoclasts were the primary cells responsible for bone resorption, in 1962, a study conducted by Baud utilized electron transmission microscopy to observe and demonstrate that osteocytic lacunae, the small spaces in which osteocytes are housed within bone, exhibited irregular borders that bore resemblance to the characteristics associated with osteolytic activity [32,33]. According to Neuman and Ramp [34], calcium released through osteoclastic bone resorption accounted for only about 0.1% of the total calcium release. This suggested that osteoclastic resorption alone could not entirely explain the systemic calcium regulation in bone metabolism. Later, based on these findings, it was discovered that when parathyroid hormone was administered to rats that had undergone thyroparathyroidectomy (surgical excision of both the thyroid and parathyroid glands), the calcium released into the bloodstream did not originate from the superficial zone where osteoclasts are active, but rather from the deeper region where osteoclasts are absent. This observation supported the concept of osteocytic osteolysis. Using transmission electron microscopy on osteocytes from rats administered PTH revealed increased lysosomes, endoplasmic reticulum, and Golgi apparatus, along with lytic changes in the walls of lacunae [33].

Mechanosensation: In general, osteoclasts and osteoblasts are believed to control functional adaption, but the mechanism through which these cells receive instructions to do so remains unclear. These cells act on the bone tissue’s surface, while mechanical loads result in strains across the tissue or even deeper. Hence, osteoblasts and osteoclasts must be informed about the local tissue requirements, and the detection of an abnormal strain is most effectively carried out by cells dispersed within the bone tissue—osteocytes. We can therefore say that osteocytes play a significant role in the anabolic response triggered by mechanical stimuli. They have the capacity to perceive physical changes stemming from mechanical strain on the bone and translate these changes into signals that can prompt either bone resorption or bone formation. This process is commonly referred to as “mechanotransduction” [35,36,37]. In this context, osteocytes function as the sensors of abnormal mechanical loads, and they can potentially guide osteoblasts to adjust their activity. Osteoblasts then have the capability to regulate osteoclast activity and thereby influence local bone resorption. The critical question is how osteocytes acquire information about their mechanical environment and how they subsequently communicate instructions to osteoblasts [30]. This question remains unanswered due to the absence of a clear comprehension regarding the transduction of mechanical stimuli into a cellular signal in vivo. In this paragraph, we have described that osteocytes can detect mechanical changes and send signals that lead to either bone formation or resorption, but we still do not fully understand how osteocytes receive information about their mechanical environment. The prevailing assumption is that osteocytes respond to strain, a slight deformation of the calcified bone matrix, or related consequences, such as fluid flow or electrical effects. However, definitive experimental evidence to support this theory is still lacking [22].

Osteocytes as regulators of bone remodeling: Osteocytes are involved in bone formation regulation, notably through their influence on the Wnt/β-catenin complex, a critical signaling pathway that governs bone formation in osteoblasts. This complex stimulates osteoblastogenesis through the activation of β-catenin. This occurs when Wnt binds to its receptor complex, comprising Frizzled and LRP5/6 (this process was described in more detail in the previous subsection concerning the communication between osteoclasts and osteoblasts). Osteocytes are able to inhibit bone formation by releasing sclerostin and Dkk-1 (Dickkopf protein-1), both of which bind to LRP5/6, acting as inhibitors of the Wnt/β-catenin signaling pathway. Furthermore, sclerostin stimulates osteocyte-derived RANKL, which supports the process of osteoclast formation. Thus, apart from releasing sclerostin and Dkk-1, osteocytes are also considered to play a role as the primary source of RANKL and OPG [30].

Sclerostin, which is produced by osteocytes and encoded by the SOST gene, acts as a negative regulator of bone formation by acting as an inhibitor of the canonical Wnt signaling pathway. Intermittent PTH treatment can reduce SOST expression and increase bone formation. The use of a sclerostin antibody, Scl-Ab, leads to enhanced bone formation either by attracting new osteoblasts to dormant surfaces or by directly activating quiescent bone-lining cells. Hence, the use of a sclerostin antibody, Scl-Ab, has emerged as a promising approach for osteoporosis treatment [38].

Osteocytes as endocrine cells: Certain researchers have conceptualized the osteocyte network as an endocrine organ that regulates phosphate metabolism. We have already described how osteocytes contribute to the localized control of bone resorption through RANKL or through sclerostin and Dkk-1. Additionally, osteocytes produce systemic factors that can act as hormones, such as FGF-23, DMP-1 (dentin matrix protein-1), PHEX (phosphate-regulating genes with homologies to endopeptidases on the X chromosome), or MEPE (matrix extracellular phosphoglycoprotein) [30]. FGF-23 is a hormone primarily released by osteocytes and osteoblasts. The pivotal function of FGF-23 is maintaining bone metabolism and the balance of mineral ions, especially phosphate levels in the body [39]. Furthermore, FGF-23 inhibits bone mineralization, and overexpression of FGF-23 can lead to a condition known as phosphaturia, where there is excessive loss of phosphate through the kidneys [40,41]. The consequence of this imbalance is the development of rickets and osteomalacia (both of them are characterized by weakened and poorly mineralized bones due to the inadequate incorporation of minerals into the bone matrix). On the other hand, the loss of FGF-23 in mice (FGF-23^−/−^ mice) exhibits elevated levels of serum phosphate, calcium, and 1,25-(OH)_2_D. Interestingly, despite the high systemic levels of mineral ions, these mice experience severe defects in skeletal mineralization (and other phenotypic manifestations, such as infertility, extensive soft tissue calcifications, muscle wasting, and profound growth retardation). The exact cause of this abnormality remains largely unexplained. Also, researchers have found a notable increase in the expression and accumulation of osteopontin (one of the inhibitors of bone mineralization) in the bones of FGF-23^−/−^ mice. This fact, coupled with the presence of high systemic levels of mineral ions and the characteristic abnormalities in bone mineralization, can provide a possible explanation for the impaired mineralization observed in the bones of FGF-23^−/−^ mice. By generating FGF-23^−/−^/OPN^−/−^ double-knockout mice, it was found that these mice exhibited improved overall health and size compared to FGF-23^−/−^ mice. Biochemical analysis of serum showed that the double-knockout mice had similarly elevated ion levels (hypercalcemia, hyperphosphatemia, and elevated serum 1,25-(OH)_2_D). However, µCT analysis confirmed that trabecular bone volume fraction in double-knockout mice was restored, exceeding those of wild-type and OPN^−/−^ mice (compared to FGF-23^−/−^ mice, trabecular bone volume fraction was significantly reduced) [7,42].

Bone micro-damage repair: Osteocyte apoptosis is believed to serve as a trigger mechanism for local bone remodeling and micro-crack repairing by attracting osteoclasts toward the sites of bone damage. When micro-damage forms, alternations in bone fluid composition and reduced oxygen levels occur. Osteocytes are able to detect these changes, and as a result, they undergo apoptosis, and neighboring osteocytes release signals (e.g., TNF-α, IL-6, IL-11) that attract osteoclasts to remove the damaged bone [30].

Bone-lining cells derive from osteoblasts and cover the bone surfaces where active bone resorption or bone formation does not actively occur. They have flat or slightly ovoid nuclei and few organelles, like rough endoplasmic reticulum and Golgi apparatus. Normally, they show little sign of synthetic function, and their activity depends on the bone’s condition, and they can become more active and enlarge. They also seem to prevent direct interaction between osteoclasts and the bone matrix when active resorption is not required [16].

#### 2.2.4. Communication between Bone Cells

It is important to note that osteoblasts and osteoclasts communicate with each other, and the dynamic coupling between bone resorption and bone formation is necessary for regulating and coordinating their activities and maintaining bone homeostasis, which refers to the balanced and stable state of bone tissue. Three primary mechanisms and some examples of signaling molecules and factors through which they communicate are [43]:

Ephrin-B2/ephB4: Ephrin-B2 is a membrane-bound ligand on osteoclasts, and ephB4 is its corresponding receptor on osteoblasts and osteocytes. When the ligand ephrin-B2 binds to the ephB4 receptor, this interaction affects bone resorption and regulates osteoblast function and bone formation. The forward signaling, observed in vitro, starts with the expression of ephB4 and promotes the expression of genes related to osteogenic differentiation, whereas reverse signaling, starting with Ephrin-B2 expression, initiates negative feedback and inhibits osteoclast differentiation [18,43,44,45].

Semaphorin 3A: semaphorins are a family of membrane-associated proteins that have been shown to influence bone remodeling and osteoblast–osteoclast communication. Semaphoring 3A is considered to be a diffusible axonal chemorepellent (in layman’s terms, chemorepulsion is also called negative chemotaxis, which means that when axons encounter the molecule, it signals them to change their direction) that plays a significant role in nervous system development. In the context of bone modeling and remodeling, semaphorin 3A is mainly produced by osteoblasts (or osteoblast lineage cells), and its receptor Neuropilin-1 (Nrp1) is expressed by osteoclast precursors. It acts as a chemo-repellent for osteoclast precursors expressing Nrp1, and this mechanism leads to the inhibition of bone resorption and the promotion of bone formation [18,46].

FAS ligand (FASL)-FAS: estrogen has been shown to promote osteoclast apoptosis, and one of the mechanisms through which estrogen induces osteoclast apoptosis is by induction of FASL (Factor associated suicide ligand, also known as CD95 or APO-1). First of all, estrogen binds to estrogen receptors on osteoblasts, and this binding induces the osteoblasts to produce and release FASL. The FASL released by osteoblasts then interacts with its receptor on the surface of pre-osteoclasts, FAS, and this binding leads to the activation of apoptosis in these osteoclast precursors. With FASL knocked out in osteoblasts, the interaction between FASL and FAS on pre-osteoclasts is disrupted, resulting in decreased osteoclast apoptosis and excessive bone resorption [18,43].

##### Osteoblast-Secreted Factors

Macrophage colony stimulating factor (M-CSF), also known as colony-stimulating factor 1 (CSF-1): M-CSF is a cytokine that plays a crucial role in the differentiation, survival, and proliferation of monocytes, macrophages, osteoclasts, and their precursor cells. This growth factor is secreted from osteoblasts and binds to its receptor, c-FMS, on the surface of osteoclast precursor cells. This binding contributes to forming multinucleated mature osteoclasts and maintaining the functional lifespan of osteoclasts. The absence of M-CSF in op/op mice (osteopetrotic mice with a mutation in the csf1 gene that leads to the expression of non-functional M-CSF) results in disruption of the normal bone remodeling process—since osteoclasts rely on M-CSF for their development and activation, their deficiency leads to the observed osteopetrotic phenotype in these mice [18,43]. In addition to the severe deficiency of osteoclasts, affected op/op mice also suffer from an extremely low total number of mononuclear phagocytes. Based on these observations, it is reported that M-CSF is involved in the regulation of certain tissue macrophage populations, but it is not the sole factor inducing and governing macrophage differentiation. Surprising findings emerged when studying the osteopetrotic op/op mice—contrary to the initial expectations, the op/op mice showed that the number of blood monocytes, a type of mononuclear phagocyte, was normal despite the mutation in the CSF-1 gene. Instead, the functions and numbers of several tissue macrophage populations were found to be changed (specifically, there was a significant reduction in macrophage numbers in various tissues such as the kidney, liver, or dermis) [47,48].

Receptor Activator of NF-κB (Nuclear Factor-Kappa B) Ligand (RANKL): RANKL is a membrane-bound cytokine and a member of the tumor necrosis factor (TNF) superfamily. It is primarily produced by osteoblasts. RANKL is not only an osteoclast differentiation factor but also serves as an osteoprotegerin ligand OPGL. When bone remodeling is required, osteoblasts express RANKL on their cell surface. RANKL subsequently binds to its receptor, RANK (Receptor Activator of Nuclear Factor Kappa-B), present on osteoclast precursor cells. This binding (along with co-stimulatory signaling that leads to Ca^2+^ oscillation, which is essential for the production of a transcription factor NFATc1) induces differentiation of osteoclast precursors into mature osteoclasts and is necessary for the proper functioning of mature osteoclasts in bone resorption. If RANKL is knocked out in mice, it leads to a phenotype characterized by osteopetrosis [18,43,49].

**Figure 7:**
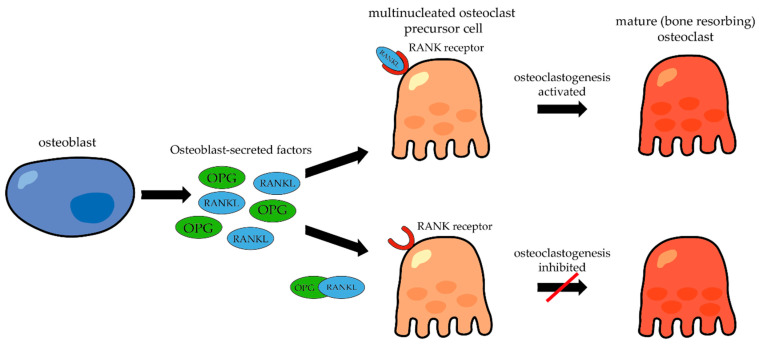
Effect of RANKL on osteoclastogenesis.

Osteoprotegerin OPG (osteoclastogenesis inhibitory factor): osteoprotegerin is a member of the tumor necrosis factor receptor superfamily (TNFR). It is named like that to reflect its role in protecting bone against bone loss. OPG is produced by various cell types, including osteoblasts and cells in the liver, spleen, or heart. This glycoprotein lacks transmembrane and cell-association signals, suggesting that it likely acts in the extracellular environment. Overexpression of OPG leads to increased bone density (osteopetrosis) because of inhibition of the differentiation of osteoclasts. OPG knock-out mice, on the other hand, exhibited severe bone loss and osteoporosis. These characteristics highlight its potential as a treatment for osteoporosis or bone loss due to other pathological changes [18,43,50]. The balance between RANKL and OPG expression is crucial for the regulation of osteoclastogenesis. For example, when the RANKL/OPG ratio increases (more RANKL or less OPG expression), it favors osteoclastogenesis and bone resorption. Conversely, when the RANKL/OPG ratio decreases (less RANKL or more OPG expression), it inhibits osteoclastogenesis and favors bone formation (Figure 7) [51]. If RANKL binds to its receptor, RANK, which is expressed on the surface of osteoclast precursor cells, this binding triggers signaling pathways that activate osteoclastogenesis and leads to the differentiation of osteoclast precursors into mature osteoclasts. Furthermore, RANKL not only acts as a factor inducing osteoclast differentiation but also serves as a ligand for osteoprotegerin (OPG), which serves as an inhibitor of osteoclastogenesis [52].

Leucine-rich repeat-containing G-protein-coupled receptor 4 (LGR4, also known as GRP48): LGR4 is a member of the G-protein-coupled receptor family, and it was found to be expressed in osteoblasts, among others. LGR4 plays a dual role in regulating both osteoclastogenesis and osteoblastogenesis, thereby impacting bone homeostasis. It competes with RANK for binding to RANKL, which reduces the availability of RANKL to interact with its receptor on osteoclast precursor cells. This competition leads to the inhibition of osteoclast differentiation and maturation. LGR4 also activates the canonical Wnt/β-catenin signaling pathway, which is essential for osteoblastogenesis and bone formation. When LGR4 is deleted or knocked out in mice, embryonic bone formation is delayed, impaired differentiation and mineralization of osteoblasts occurs, and this altered bone development leads to many bone-related pathological conditions [51,52,53,54].

The Wnt family: a group of signaling molecules that are involved in various developmental processes, including embryogenesis, as it regulates cell fate determination, postnatal development, and so on. Wnt proteins also play a significant role in regulating bone homeostasis. They can bind to two receptor complexes on the cell surface, leading to the activation of two main pathways: the β-catenin-dependent canonical pathway and the β-catenin-independent noncanonical pathway. The canonical pathway is distinguished by its reliance on the stabilization of β-catenin, whereas the non-canonical pathway operates independently of β-catenin.

**Figure 8:**
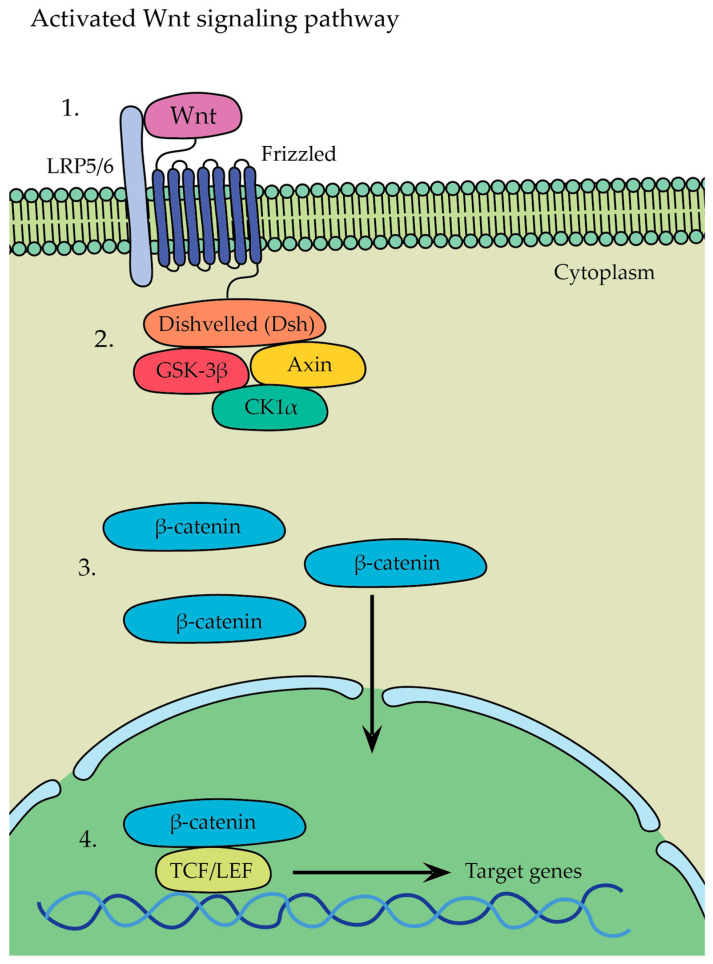
Activated Wnt signaling pathway: (1) the Wnt ligand binds to Frizzled and LRP5/6 receptors; (2) Frizzled interacts with Disheveled (Dsh); (3) β-catenin translocates into the nucleus; (4) β-catenin forms a complex with various enhanceosome proteins.

- β-catenin-dependent canonical pathway (Figure 8)—in this pathway, Wnt ligands bind to a receptor complex consisting of Frizzled and LRP5/6, which leads to the initiation of a signaling cascade where glycogen synthase kinase 3 stabilizes β-catenin → as a result, β-catenin is no longer targeted for degradation and accumulates in the cytoplasm, whereupon it translocates from there into the nucleus, leading to the activation of gene transcription program and initiation of the expression of target genes involved in various cellular processes, such as osteoblastogenesis, osteocyte formation, and bone tissue development.
- β-catenin-independent non-canonical pathway—Wnt5a, expressed in osteoblast-lineage cells, is a main ligand for non-canonical Wnt signaling that binds to its receptor complex of Frizzled and ROR2, present on the surface of osteoclasts, and it has been shown to activate both the Wnt-Ca^2+^ and Wnt-JNK pathways; Wnt5a was found to enhance RANKL-induced osteoclastogenesis due the upregulation of RANK expression in osteoclasts and activation of the Jun-N-terminal kinase MAPK pathway (JNK) [18,55,56].

The Wnt ligand binds to Frizzled and LRP5/6 receptors located on the surface of osteoblasts (Figure 8(1)). Frizzled interacts with Disheveled (Dsh), which inhibits GSK-3β activity, preventing the phosphorylation and degradation of β-catenin (Figure 8(2)). The inhibition of GSK-3β by Dsh disrupts the GSK-3β/Axin/CK1α destruction complex and recruits it to the membrane, leading to the accumulation of β-catenin in the cytoplasm of the osteoblast. This accumulation allows β-catenin to translocate into the nucleus (Figure 8(3)). Inside the nucleus, β-catenin forms a complex with various enhanceosome proteins, such as the LEF/TCF family of DNA-bound transcription proteins, initiating the transcription of specific target genes involved in osteoblast differentiation and bone formation (Figure 8(4)) [57].

1. Osteoclast-Secreted Factors

Atp6v0d2 (also known as d2 isoform of vacuolar ATPase, or v-ATPase, V0 domain): Atp6v0d2 is expressed in osteoclasts, and its role is to regulate the function and assembly of the v-ATPase complex in osteoclasts because proper v-ATPase activity is essential for osteoclast function and bone resorption. When Atp6v0d2 is deactivated in mice, there is a significant increase in bone mass. This is attributed to impaired osteoclasts and enhanced bone formation. According to Lee et al., the deficiency of Atpv0d2 did not impact the differentiation of osteoclasts or their v-ATPase activity. Rather, Atp6v0d2 played a significant role in facilitating efficient pre-osteoclast fusion. The findings from Atp6v0d2-deficient mice, despite showing a marked increase in bone formation, indicate that Atp6v0d2 does not directly influence osteoblast differentiation or the expression of osteoblast differentiation markers. In this context, it appears that Atp6v0d2′s role in bone formation may be indirect or mediated through other pathways that indirectly regulate osteoblast activity [43,58].

Semaphorin 4D: Sema4D is an axon guidance molecule belonging to the semaphoring family of cell-signaling molecules. Sema4D is involved in many cellular processes; apart from suppressing osteoblast differentiation, it plays a pivotal role in immune regulation and cancer bone metastases. It is expressed on osteoclasts and binds to its receptor Plexin-B1 on the surface of osteoblasts. After that, Plexin-B1 forms a receptor complex with the tyrosine kinase receptor ErbB2 (erythroblastic leukemia viral oncogene homolog 2) in osteoblasts. The binding of Sema4D to Plexin-B1 induces the phosphorylation of ErbB2, which activates its kinase function in osteoblasts. The Sema4D-Plexin-B1-ErbB2 complex induces activation of GTPase RhoA. Activated RhoA then mediates the downregulation of insulin-like growth factor-1 (IGF-1) signaling in osteoblasts. By inhibiting IGF-1 signaling, osteoblast differentiation is suppressed. The absence of Sema4D in mice leads to disruption in the normal signaling pathway that suppresses osteoblast differentiation. As a result, osteoblasts in Sema4D-deficient mice undergo enhanced differentiation, leading to increased bone formation and higher bone mass. Similar phenotypes are also observed in Plexin-B1-deficient mice [18,43,59].

Sphingosine 1-phosphate (S1P): S1P is a lipid molecule formed by the phosphorylation of sphingosine by sphingosine kinase (SPHK) enzymes. When S1P binds to its receptor located on the surface of osteoblasts, the expression of RANKL is upregulated, resulting in the promotion of osteoclast differentiation. In addition to its function in enhancing osteoclast formation, S1P also appears to play a role in promoting osteoblast migration, survival, and precursor recruitment [18,60].

Collagen Triple Helix Repeat Containing 1 (CTHRC1): CTHRC1 is a protein that is predominantly expressed by osteoclasts during their bone-resorbing activity. Research has shown that the expression of the CTHRC1 gene can be influenced by exposure to hydroxyapatite and calcium. Furthermore, CTHRC1 has been shown to stimulate osteoblast differentiation, promoting bone formation. To understand the role of osteoclast-derived CTHRC1 in bone remodeling, the mice were bred to gain a conditional CTHRC1 knockout allele. According to these findings, they showed a low bone mass phenotype similar to osteoporosis, with reduced trabecular number and thickness [18,61].

Complement component C (C3): osteoclast-derived C3 plays a crucial role in bone homeostasis, serving as a vital link between osteoclasts and osteoblasts. It has a significant effect on bone cell growth, differentiation, and development. Its bioactive fragment, C3a, enhances osteoclast formation. On the other hand, its receptor, C3aR, is expressed during the differentiation of human mesenchymal stem cells (MSCs) into mature osteoblasts, suggesting that C3a may play a role in both osteoblast differentiation (promoting osteoblastogenesis) and bone resorption. C3 deficiency has been associated with certain autoimmune conditions, such as systemic lupus erythematosus [43,62].

2. Examples of other factors influencing either osteoblast or osteoclast behavior

Sclerostin: The SOST gene, responsible for producing sclerostin, is predominantly expressed in osteocytes. When sclerostin interacts with the LRP5/6 and Frizzled co-receptors on the surface of osteoblasts, the Wnt/β-catenin signaling pathway is inhibited. This inhibition leads to a decrease in osteoblast differentiation, proliferation, and activity, ultimately resulting in reduced bone formation by osteoblasts. Sclerosteosis is an extremely rare genetic disorder that is caused by mutations in the SOST gene and is characterized by progressive thickening of the bones, enlargement of the skull, and so on. Foraminal stenosis can even compress cranial nerves and cause facial paralysis [63].

MicroRNAs: microRNAs are a class of short, single-stranded, non-coding RNAs that have significant implications in various diseases, including cardio-vascular diseases, cancer, and osteoporosis. On the other hand, research has highlighted the potential of microRNAs as valuable tools for disease diagnosis or biomarkers. For example, osteoclast-derived miR-217-3p has been found to negatively regulate the process of osteoblastogenesis and osteoblastic bone formation towards adipogenic differentiation [43,64].

Parathyroid hormone (PTH): this is an endocrine hormone secreted by the parathyroid glands. Its primary role is to maintain calcium metabolism in the body and help to regulate bone remodeling. When PTH is injected daily as a treatment for osteoporosis, it has a positive effect on bone health by stimulating bone formation. However, it is important to note that continuous infusion of PTH can have detrimental effects on bone health—extended exposure to high levels of PTH can lead to severe bone loss. Thus, since PTH has dual effects on bone, as it stimulates both bone formation and bone resorption, the resulting net effect of PTH on bone mass, either anabolic or catabolic, depends on the duration and periodicity of its exposure. Continuous treatment results in catabolic effects on the skeleton, while intermittent PTH administration results in osteoanabolic effects. In the context of bone resorption, PTH injection can have varying effects on the ratio of two key regulators of bone resorption: RANKL and OPG. For example, continuous PTH infusion in rats has been shown to lead to a decrease in OPG and an increase in RANKL mRNA levels, and this shift favors the differentiation of osteoclasts and recruitment of osteoclast precursor cells, leading to an increase in the number and activity of mature osteoclasts. On the other hand, intermittent PTH administration allows for periods of bone formation to occur, which may counterbalance the effects of bone resorption [43,65,66].

Transforming Growth Factor β1 (TGF-β1): in bone metabolism, TGF-β1 is a key player, influencing both osteoblasts and osteoclasts. It helps to regulate bone remodeling and maintain a delicate balance between the processes of bone resorption and bone formation throughout life. Normally, TGF-β1 exists in an inactive form, bound to a latent-associated peptide (LAP). In the acidic milieu created by osteoclasts during bone resorption, the low pH might disrupt the non-covalent bonds between LAP and TGF-β1. Once activated, TGF-β1 attracts bone mesenchymal lineage cells to the resorptive surfaces. Subsequently, these recruited cells undergo differentiation into osteoblasts, the bone-forming cells [43,67].

#### 2.2.5. Osteon Structure (Haversian System)

There are two types of bone tissue: solid cortical bone, which provides mechanical strength and most of the support for the body, and spongy trabecular bone, which is less dense than cortical bone and has a honeycomb-like structure. Osteons are the fundamental structural and functional units of cortical bone. They can be categorized into two main types [68]:

- Primary osteons—they are situated near primary bone, and they usually contain fewer circular lamellae compared to the secondary osteons,
- Secondary osteons—also known as Haversian systems, they are the primary functional units of cortical bone that develop from primary osteons during bone remodeling. Each secondary osteon has concentric lamellae arranged around the central canal that houses blood vessels, nerves, and connective tissue, and these systems are distinguished from each other by clear cement lines that define secondary osteon boundaries.

Because of the ongoing bone-remodeling process, osteons are constantly being renewed. This leads to the presence of osteons at various levels of calcification within the adult compact bone. In general, osteons in the early stages of development are less mineralized and appear more transparent to X-rays compared to fully mineralized osteons that have reached their final stage of formation [69]. When compared to trabecular bone, osteons carry out better physiological functions and dynamic adaptability. Within an osteon’s structure, both osteocytes and the lacunocanalicular network LCN play pivotal roles in sensing and transducing mechanical stress, rendering the bone highly responsive to external stimuli. Furthermore, the concentric lamellae enhance bones’ resilience and capacity to endure mechanical loads. Another key feature of osteon is the presence of the Haversian canal. This central canal houses a network of blood vessels and nerve fibers, providing essential nutrients and sensation to the osteocytes nestled within the lamellae. Additionally, the Haversian canal also serves as a conduit for transporting both osteoclasts and osteoblasts and thus enables cortical bone remodeling [68].

As already mentioned in the chapter called Cells of bone, osteocytes are the most common cell type in bones and represent fully matured bone cells that are embedded within the mineralized bone matrix. Osteocytes are distinctive in their stellate-shaped morphology, with numerous dendrites extending from their cell body. These dendritic projections enable extensive cellular connections and communication within the bone tissue. Osteocytes are asymmetrically distributed within osteons—osteocyte density reaches its peak in proximity to the cement line. This fact suggests a potentially important role in maintaining bone integrity and participating in mechanosensory functions [68].

When it comes to the formation of dendritic processes, E11/gp38 may induce and regulate the formation of these projections. This protein is selectively associated with osteocytes (not with osteoblasts) and appears in the developing dendritic processes of the osteocytes. Furthermore, E11 expression responds to mechanical loads, and alterations in the complexity of osteocyte dendritic networks can impact the mechanical characteristics of bone as a whole, the bone’s ability to respond to mechanical stimuli and carry out its role in bone remodeling, etc. [70,71]

The lacunocanalicular network represents the intricate network formed by the spaces containing osteocyte cell bodies (lacunae) and their dendrites (canaliculi). It serves as a conduit for nutrient transportation and waste removal. Also, the fluid-filled spaces within the lacunocanalicular network enable osteocytes to detect mechanical cues, translating them into cellular signals that initiate cellular responses. This response involves the release of signaling molecules that influence bone remodeling, mineral homeostasis, etc. [68,72]

**Figure 9:**
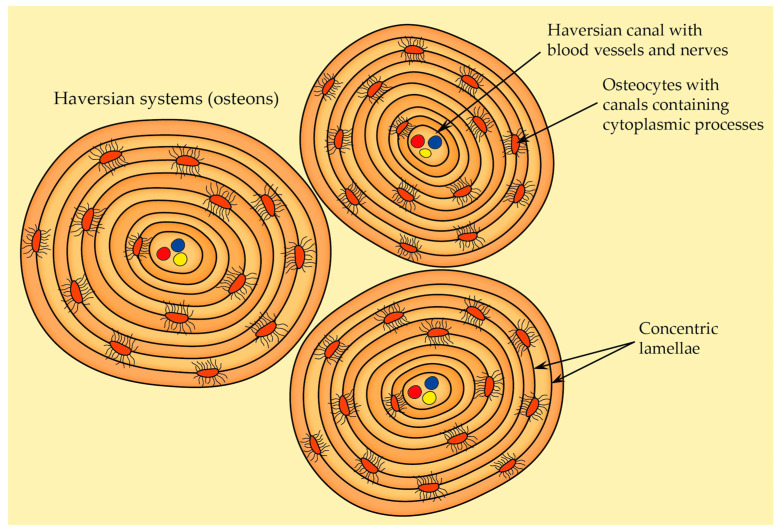
The structure of Haversian system (osteon). Each osteon contains a single central Haversian canal encircled by concentric lamellae.

The most prominent and recognizable characteristic of osteons is the arrangement of concentric lamellae (Figure 9). A mature secondary osteon generally consists of four to eight layers of these lamellae. They can be described as circular layers of bone tissue, and when observed under polarized light microscopy, the lamellae within osteons exhibit a distinct alternating pattern in appearance. Both lamellar bone and the osteonic bone (the type of bone found within osteons) reveal an alternation between isotropic and anisotropic lamellae. This phenomenon is a result of the intricate organization, thickness, and alignment of collagen fibers within bone tissue. The gaps between the Haversian systems are occupied by interstitial lamellae [68,69].

A continuous structure known as the cement or reversal line encircles the newly formed osteons, maintaining a distinct boundary between them and the interstitial bone. Cement lines serve as the distinctive features that distinguish primary osteons from secondary ones. They are identified as thin discontinuous lines formed due to the actions of a basic multicellular unit (BMU) (groups of cells consisting of different cell types, mainly osteocytes, osteoblasts, and osteoclasts, coordinating in order to facilitate bone replacement [39]) on resorbed surfaces of bone. Cement lines are highly mineralized and have low collagen content. Nonetheless, they appear to have notably lower levels of calcium and phosphorus (their Ca/P—or calcium to phosphorus—ratio is lower compared to the surrounding bone matrix) while containing higher levels of sulfur. Also, there exists a positive correlation between the mineralization level of the cement line and that of the osteon. As the mineralization of the osteon increases, the contrast in mineralization between the cement lines and the osteon lamellae decreases. This suggests that the mineralization of the cement line relies on the mineralization of the osteon and is regulated by aging. Cement lines play a significant role in influencing microcrack propagation within bone. This is attributed to their elevated mineralization level, notable mechanical resilience, and unique location at interfaces [68,69].

#### 2.2.6. Bone Blood Supply

The long bone is normally supplied by four types of arteries: nutrient arteries, periosteal arteries, metaphyseal arteries, and epiphyseal arteries. One or two primary nutrient arteries penetrate the shaft through openings called nutrient foramina, which lead to nutrient canals. Inside these canals, the nutrient arteries do not divide, but within the medullary cavity, they divide into ascending and descending branches. These branches subsequently divide into smaller vessels, which can eventually reach the endosteal surface. Close to the epiphyses, these vessels can anastomose with terminal branches from metaphyseal (originating from nearby systemic vessels) and epiphyseal arteries (originating from networks of blood vessels that are formed around the bone surfaces adjacent to the joints) [1].

Haversian canals are cylindrical spaces that are enclosed by the innermost layer of osteon lamellae and run parallel to the primary axis of an osteon (this inner surface of the innermost lamella is covered by flat cells surrounded by densely packed collagen fibers). These canals house nerve fibers and blood vessels, facilitating the nourishment of osteocytes. Typically, one to two blood vessels can be observed within a Haversian canal. Additionally, it serves as the initial location for the remodeling of osteons [68].

Volkmann’s canals are perpendicular to the main bone axis; they are similar in size to Haversian canals but are present in fewer numbers. They connect to Haversian canals [68].

### 2.3. Overview of Bone Types and Their Organization

Woven bone lacks the distinct layers seen in lamellar bone and is notable for its random arrangement of type I collagen fibers. This type of bone is the initial form that appears during embryonic development and serves as a framework—subsequently, layers of lamellar bone are added onto this framework. Thus, woven bone is typically temporary, and in adults, it is replaced by the more organized lamellar bone, with a few exceptions, such as near the sutures of the skull and within certain tendon attachments [12,73]. There are two different groups of osteoblast cells contributing to the creation of woven and lamellar bone. The first, mesenchymal osteoblasts, surround themselves with collagen fibers arranged randomly, forming woven bone. The second group, surface osteoblasts, arrange themselves in a linear pattern on the surface of woven bone, producing parallel-fibered lamellar bone. This whole process can be divided into the following four stages [73]:

1. Stage I—the initial differentiation of pre-osteoblasts from their stem (mesenchymal) cells.
2. Stage II—mesenchymal osteoblasts encircle themselves with matrix fibers oriented in a random manner.
3. Stage III—the woven matrix serves as a scaffold where surface osteoblasts start synthesizing bone with a parallel-fibered lamellar structure.
4. Stage IV—gradual reduction in woven bone and progression from woven to lamellar bone.

Lamellar bone, also known as secondary or mature bone, earns its name because it replaces the woven or primary bone, which is gradually resorbed after birth through bone remodeling (however, under certain pathological circumstances, e.g., bone tumors, woven bone can reappear). It constitutes the majority of the adult skeleton, and the arrangement of bone layers (lamellae) differs depending on the location [1,69]:

- In the trabecular bone and on the outer (periosteal) and inner (endosteal) surfaces of cortical bone, a few lamellae shape continuous circular layers that are approximately parallel to the bone surface.
- In the central regions of cortical bone, the lamellae form concentric cylinders and encircle Haversian canals, and this organization enhances the resilience of lamellar bone since the interfaces between lamellae can stop the expansion of cracks (and more energy is necessary to propagate cracks extensive enough to result in bone fractures).

About 80% of the entire bone mass is attributed to cortical (or compact) bone, which plays a crucial role in maintaining the mechanical strength of bone. The functional units of cortical bone are known as osteons or Haversian systems (which have already been described in previous chapters), and they constitute most of the cortical bone. Furthermore, multiple external circumferential lamellae are situated just beneath the periosteum, while fewer inner circumferential lamellae encircle the marrow cavity. These lamellae in the outer and innermost regions of cortical bone provide support and reinforcement to the middle region [1,12].

Trabecular (cancellous) bone, in contrast to the cortical bone, is notably porous, heterogeneous, and anisotropic. It is located at the epiphyses of long bones and within vertebral bodies and serves as the primary load-bearing component. Additionally, it is organized to optimize the transfer of loads from joints (or rather articular surface) to the cortical bone of long bones. Furthermore, it is covered in endosteal tissue due to its proximity to marrow cavities. While thick trabeculae and areas near compact bone might contain tiny osteons, blood vessels generally do not run within trabeculae [74]. Approximately 20% of bone mass is trabecular bone, but the proportion of cortical and trabecular bone differs significantly among different bones (for instance, the ulna is composed of 92% cortical bone and only 8% trabecular bone, whereas vertebrae comprise more or less 62% cortical bone and 38% trabecular bone) [13].

When viewed in cross-section, the end of a long bones like the femur presents a solid cortical shell encasing a porous, cancellous interior. But flat bones like the scapula or calvaria exhibit a layered composition: dense cortical layers on their external surfaces and a thin, supportive cancellous structure within [75].

### 2.4. Overview of Macroscopic Properties of Bone

#### 2.4.1. Diaphysis and Epiphyses

**Figure 10:**
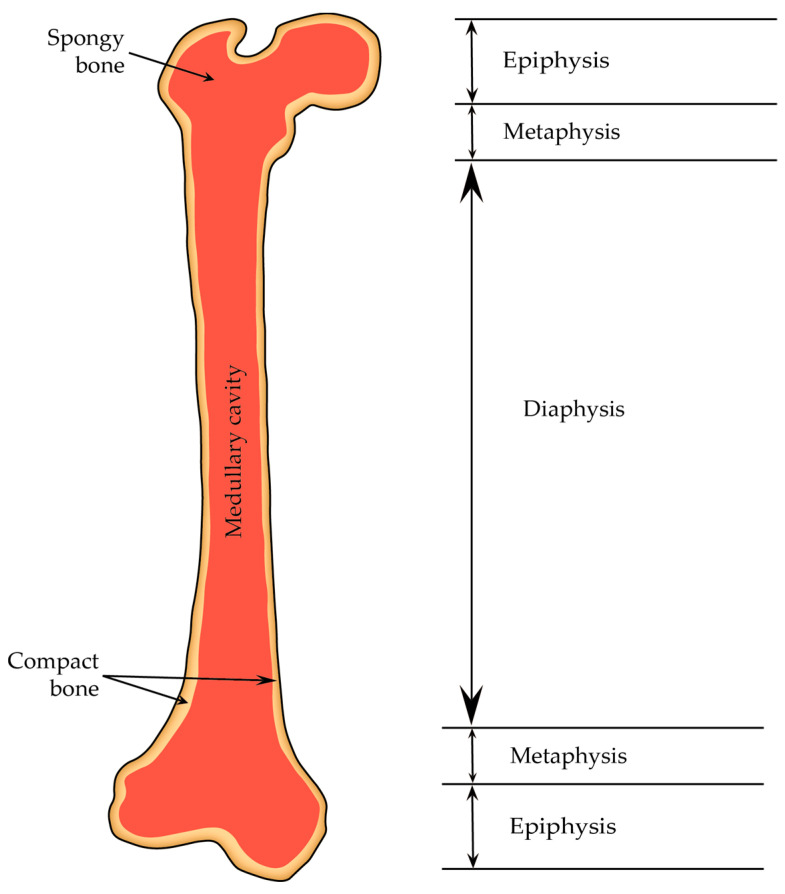
Structure of a long bone. The distal and proximal ends (epiphyses) are separated from the shaft (diaphysis) by metaphyses.

A typical adult long bone is divided into the following parts: a central cylindrical core known as the diaphysis and two wider ends called the epiphyses (they are wider than the diaphysis because they encompass joints covered by articular cartilage, and sustaining consistent loads require more substantial areas of this cartilage than bone alone). The diaphysis is linked to each epiphysis through conical regions referred to as the metaphysis (Figure 10) [13].

The diaphysis primarily consists of cortical bone, whereas the epiphysis and metaphysis are predominantly composed of trabecular bone with a thin outer layer of cortical bone. Moreover, the growing animals have a layer of hyaline cartilage referred to as the growth plate metaphyseal complex localized between the epiphysis and metaphysis. This growth plate, or epiphyseal plate, along with the surrounding cancellous bone, forms a region where bone formation and cortex elongation take place. In adulthood, the growth plate is replaced by cancellous bone, resulting in the fusion of the epiphysis with the metaphysis [13].

#### 2.4.2. Periosteum and Endosteum

When we talk about bone tissue, we distinguish two main surfaces: periosteal and endosteal. The endosteal surfaces can be further divided into the intracortical (Haversian or osteonal), endocortical, and cancellous surfaces (this surface area of cancellous bone accounts for over 60% of the total bone surface) [13].

The periosteum is composed of two layers: an outer fibrous membrane (characterized by irregular and dense connective tissue that consists of fibroblasts, collagen and elastin fibers, and a network of nerves and microvessels) and an inner cellular layer, or cambium layer, that directly contacts the bone surface (composed of osteoprogenitor cells; hence, the inner cellular layer is also referred to as the osteogenetic layer. Within the inner layer, osteoblasts are present in young bones, and in the case of adult bones, osteoblasts might not be consistently present, but they become active whenever needed) [76,77]. The inner cambium layer reaches its maximum thickness in the fetus and gradually becomes thinner with age. In adulthood, the density of blood vessels and cells decreases; thus, it becomes indistinguishable from the fibrous layer above it [78].

Sharpey’s fibers are groups of collagen fibers originating from the periosteum that extend into the bone matrix, binding the periosteum firmly to the bone [77].

The periosteum is absent in regions where tendons or ligaments attach to bone, on bone ends covered by articular cartilage, on the surface of sesamoid bones, etc. Additionally, because of its rich blood supply, the periosteum contains a significant number of endothelial pericytes (cells that are in direct contact with capillary endothelial cells and can transform into various cell types, including osteoblasts). These cells could potentially act as an additional reservoir of osteoprogenitor cells [76,78].

The endosteum is a membrane that covers the inner surfaces of the bone. It lines the Haversian canal as well as all the internal spaces within the bone. The endosteum is composed of a layer of flattened osteoprogenitor cells and type III collagen fibers, also known as reticular fibers. Notably, the endosteum is thinner compared to the periosteum [77]. As already mentioned, the endosteum can be categorized into three types depending on its location: cortical endosteum (lines the bone marrow cavity), osteon endosteum (found within the osteons), and trabecular endosteum (lines the trabeculae and plays a role in the bone’s growth and development) [77].

Some functions of the periosteum and the endosteum [77]:

- Nourishment of bone tissue—blood vessels in the periosteum supply the bone’s outer surface and inner osseous tissue to some extent; these vessels are accompanied by nerves and vasomotor fibers, which control blood circulation.
- Bone growth—the periosteum is crucial for the growth of bone through surface apposition; the endosteum contributes to internal matrix formation by absorbing and depositing tissue.
- Repair—osteoprogenitor cells within the periosteum are multipotent stem cells that can prompt bone regeneration when needed, such as after a bone fracture.
- Calcium regulation—the endosteum aids in depositing calcium into the bone matrix and acts as a conduit for calcium transfer between the bone matrix and blood.

#### 2.4.3. Bone Marrow

Bone marrow is a vital organ for the process of hematopoiesis (which is the production of various types of blood cells). This continuous production of blood cells is essential for various physiological processes, including oxygen transport, immune response, and blood clotting. The bone marrow also provides a specialized and unique environment that supports the formation and development of blood cells. This microenvironment, often referred to as the hematopoietic niche, consists of various cellular and molecular components that interact to regulate the balance between stem cell self-renewal and differentiation [79].

Bone marrow is found in the cavities of bones. In newborns, all bone marrow is red and actively involved in blood cell production (red bone marrow represents myeloid tissue and is rich in hematopoietic cells). However, as a child grows, much of the red bone marrow gradually transitions to yellow marrow (it contains more fat cells—adipocytes— and fewer blood-forming cells; thus, it is considered less active in terms of blood cell production and primarily serves as a storage site for adipose tissue). Under specific circumstances, such as severe bleeding of hypoxia, the yellow bone marrow can revert to a more active red bone marrow. This adaptive response allows the body to increase its blood cell production to address the increased demand [12].

### 2.5. Bone Development and Growth

Skeletal development and bone ossification (osteogenesis) start with the aggregation of mesenchymal cells into condensed structures. These condensations serve as the initial foundation for bone formation [13]. Bone ossification specifically starts around the sixth to seventh weeks of embryonic growth and persists until approximately the age of twenty-five, although it can slightly differ from person to person [80].

There are two types of bone ossification: intramembranous and endochondral. Both processes initiate from a precursor mesenchymal tissue, but their transformations into bone are different. In intramembranous ossification, mesenchymal tissue is directly converted into bone, giving rise to flat bones of the skull and jaws, as well as the scapula or clavicle. On the other hand, endochondral ossification starts with mesenchymal tissue turning into an intermediate cartilage stage, which is eventually substituted by bone. This process contributes to the axial skeleton’s remaining parts and the formation of long bones [80].

#### 2.5.1. Intramembranous Ossification

Intramembranous ossification commences when specialized bone-forming cells known as osteoblasts differentiate from mesenchymal cells derived from the neural crest. These bone-forming cells group into clusters to establish an ossification center. Within this center, osteoblasts initiate the secretion of osteoid that is subsequently mineralized; thus, osteoblasts are trapped and transformed into osteocytes. This mineralization enlarges and leads to the fusion of nearby ossification centers. Furthermore, woven bone matrix is substituted with compact bone, surrounding an area of cancellous bone containing bone marrow and larger blood vessels. Mesenchymal areas that remain unossified become the source of the new bone’s endosteum and periosteum [12,80].

Even though intramembranous ossification is commonly linked to embryonic development, it can also take place after birth, such as during bone-healing processes. As already mentioned, the first stage of intramembranous ossification involves the gathering of mesenchymal cells. This group is also known as a bone blastema, and within this blastema, cells undergo differentiation into osteoblasts and start to produce a matrix. An essential player in this process is the transcription factor RUNX2, which guides the cells within the blastema toward the osteoblastic lineage [81].

#### 2.5.2. Endochondral Ossification

Endochondral ossification occurs within hyaline cartilage (specifically, it involves the substitution of hyaline cartilage with bone tissue) and is responsible for forming the majority of bones in the body. It initiates when mesenchymal cells differentiate into chondrocytes. As rapid proliferation of chondrocytes takes place, they secrete extracellular matrix, establishing a cartilage framework for bone formation [80]. Endochondral ossification is not exclusively confined to embryonic development; it also plays a crucial role in the healing of fractures. The whole process is under the influence of the transcription factor SOX-9 [81].

Endochondral ossification takes place in two specific regions within long bones, known as the primary (diaphyseal) and secondary (epiphyseal) sites of ossification. Bone formation begins at the primary site, while the secondary site operates independently and undergoes ossification at a later stage. A crucial element of this process is the formation of a specialized structure called the growth plate that emerges between the diaphysis and the epiphysis through the arrangement of chondrocytes at varying stages of differentiation [82]. The growth plate, also known as the epiphyseal plate, plays a crucial role in the growth of bone length. At adulthood, the epiphyseal plate eventually disappears. The process of eliminating these growth plates, often referred to as “epiphyseal closure”, takes place at different times for various bones, and by approximately the age of 20, this closure is complete across all bones [12].

**Figure 11:**
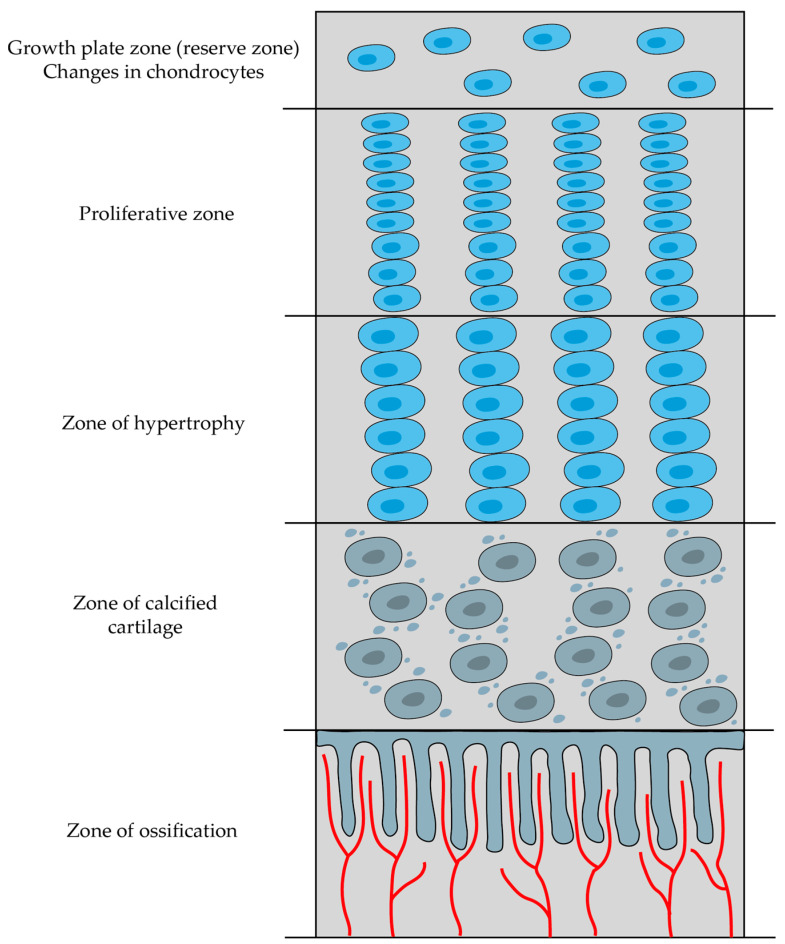
Zones of endochondral ossification.

The epiphyseal growth plate displays distinctive regions of cellular activity and is often described in overlapping but histologically distinct zones (Figure 11) [12]:

- Zone of reserve cartilage—consists of typical hyaline cartilage and serves as a reservoir of chondrocytes that can potentially contribute to growth.
- Proliferative zone—cartilage cells undergo repeated division, enlargement, and increased secretion of type II collagen and proteoglycans.
- Zone of hypertrophy—chondrocytes become enlarged and terminally differentiated; they then compress the matrix into aligned spicules and stiffen it through the secretion of type X collagen.
- Zone of calcified cartilage—chondrocytes release matrix vesicles and osteocalcin, and these substances initiate the calcification of the matrix by forming hydroxyapatite crystals.
- Zone of ossification—bone tissue first emerges in this zone; osteoblasts settle in a layer over the spicules of the calcified cartilage matrix and secrete osteoid, which subsequently transforms into woven bone (and this woven bone is later remodeled into lamellar bone).

#### 2.5.3. Appositional Growth

The expansion of the circumference of long bones does not rely on endochondral ossification. Instead, it is driven by osteoblasts that originate from osteoprogenitor cells within the periosteum, and as the bone’s circumference increases, osteoclasts in the endosteum contribute to the enlargement of the central marrow cavity. This dual activity—periosteal osteoblast-driven appositional growth and endosteal osteoclast-mediated cavity enlargement—contributes to the bone’s overall growth [12,83].

### 2.6. Bone Remodeling

Bone remodeling is a dynamic mechanism where bones change their shape due to physiological cues or mechanical pressures. This leads to a gradual adaptation of the skeleton to the various forces it encounters. Bones tend to increase in width with aging due to the addition of new bone on the outer surface (periosteal apposition) and the removal of old bone from the inner surface (endosteal resorption). Wolf’s law describes the concept that the structure of long bones adapts to handle the specific stresses they experience [11].

Bone remodeling starts before birth and continues throughout an individual’s life. It encompasses two fundamental processes: bone formation by osteoblasts and bone resorption by osteoclasts. These activities of bone formation and resorption are not always tightly coupled (except for the remodeling process itself). Even though bone formation and resorption can occur independently on different surfaces, they are not entirely independent—both processes occur concurrently throughout the skeleton and are coordinated to shape bones effectively [11,81].

Bone remodeling can be categorized as either targeted or stochastic. In targeted remodeling, a specific local signal prompts osteoclasts to initiate remodeling at a given site (this could be due to microdamage or osteocyte apoptosis, which can sometimes be related to each other when microdamage can lead to osteocyte apoptosis; other factors, such as high mineralization, might also play a role). Stochastic remodeling, on the other hand, is characterized by random osteoclast activity that does not rely on specific signals. It is believed to contribute more to calcium regulation [81,84].

#### 2.6.1. Bone Remodeling Cycle

It does not matter whether the bone remodeling is targeted or stochastic because the cellular processes involved are quite similar. This whole process can include five stages: activation, resorption, reversal, formation, and quiescence. These stages are often collectively referred to as the remodeling cycle [81,85].

##### Activation and Bone Resorption

The activation stage initiates with the recruitment and activation of mononuclear monocyte–macrophage osteoclast precursors. Multiple mononuclear cells merge to create multinucleated pre-osteoclasts. Once fully developed osteoclasts are present, bone-lining cells retract from the surface, unveiling the mineralized matrix for osteoclast interaction (without the retraction of the bone-lining cells, osteoclasts cannot attach to the bone and initiate the resorption phase). This action can be triggered either by the osteoclasts themselves as they approach the surface or by the same signals that initiated the remodeling cycle. Thus, mature osteoclasts adhere to the bone matrix by interactions between integrin receptors in their membranes and peptides rich in RGD (arginine, glycine, and asparagine) found in matrix proteins. This interaction results in the formation of annular sealing zones encircling bone-resorbing compartments positioned beneath multinucleated osteoclasts [11,81]. The process of bone resorption is discussed earlier (as well as bone formation).

##### Reversal and Formation Stage

In the reversal stage, bone resorption mediated by osteoclasts transitions to bone formation mediated by osteoblasts. The specific signals that coordinate the transition are currently not fully understood. After the resorption process, the resorption cavities house several types of mononuclear cells, such as monocytes, osteocytes, and pre-osteoblasts, that are mobilized to initiate the bone formation. It is important to note that once osteoclasts have completed the bone-resorption process, the remaining collagen fragments need to be removed. If these collagen fragments are not removed, the subsequent bone formation conducted by osteoblasts cannot proceed. It is believed that this task is carried out by a distinct type of bone-lining cell [11,81].

Osteoblasts are responsible for producing a new collagen-based organic matrix and coordinating the mineralization process of this matrix. They release tiny matrix vesicles, which accumulate calcium and phosphate, and also carry out enzymatic processes that eliminate factors that inhibit mineralization. Osteoblasts that are surrounded by the matrix transform into osteocytes [11].

#### 2.6.2. Dynamics of Bone Remodeling

The rate of bone remodeling undergoes significant fluctuations, with a rapid rate during growth that gradually decreases until peak bone mass is achieved. In adulthood, the rate is influenced by many factors, including age and genetics, as well as physical activity, nutrition, hormonal functions, and medication. Among women, remodeling activity increases during menopause due to the decrease in circulating estrogen levels (normally, estrogen impacts osteoclasts and suppresses osteoclast apoptosis). In contrast, men do not experience that dramatic increase in remodeling, which usually occurs about a decade later than observed in women [81].

In normal bone remodeling, a precise balance is upheld between bone resorption and bone formation. This balance is tightly regulated to sustain mature and healthy bone and to ensure that significant alterations in bone mass or mechanical resilience do not arise following each cycle of remodeling. However, this balance can be disrupted under certain pathological circumstances [19]. The ratio of newly formed bone in comparison to the bone that is resorbed within an individual bone-remodeling unit is termed bone (or BMU) balance. Under typical circumstances, BMU balance exhibits a slightly negative trend. However, in certain conditions like postmenopausal osteoporosis, BMU balance becomes even more negative, leading to substantial bone loss during each BMU cycle [81].

### 2.7. Pathophysiology of Major Bone Disorders

#### 2.7.1. Osteoporosis

One of the most common skeletal metabolic disorders is primary osteoporosis (aca-24). Type 1, called postmenopausal, and type 2, senile osteoporosis, are the two major types based on etiology differences, but according to more recent studies, both of these types may share a common cause in estrogen deficiency for both men and women [86,87].

The definition of osteoporosis is a decrease in bone mass, which leads to a reduction in bone strength and an increased probability of fracture. This bone mass decrease can be caused by excessive bone resorption, impaired bone formation during childhood, or impaired bone formation during remodeling processes. Even though studies show that bone remodeling during menopause is increased, the rate of resorbed bone replacement is insufficient to keep up a healthy bone structure. One of the possible causes is osteoblast malfunction due to cellular senescence and a decrease in the overall activity of the system, including its growth factors related to estrogen production reduction. The other cause is a continuous loss of bone reformation templates due to excessive resorption and accumulated damage, which may lead to perforation of trabecular plates and even removal of endosteal cortical bone [88,89].

#### 2.7.2. Osteopetrosis and Osteosclerosis

When bone resorption is insufficient in comparison to bone formation, resulting imbalance in bone turnover causes abnormal skeletal architecture. This can cause not only irregular growths to appear on the skeleton but also malformation of the skeletal structure over time, resulting in local mechanical irritations and potential tissue damage or reduction in patient mobility [90].

#### 2.7.3. Hyperthyroidism and Hyperparathyroidism

In comparison to Section 2.7.2, these disorders express themselves as an increase in bone turnover. Thyroid and parathyroid hormones stimulate bone formation and resorption. The decrease in bone mass is then reliant on the responsivity of osteoblastic cells. When the response is sufficient, bone loss will not occur. However, in severe diseases or in patients of high age, a decrease in bone mass is to be expected. In the case of hyperparathyroidism, an increase in IL-6 has been documented as a potential accompanying sign of the disorder [91,92].

#### 2.7.4. Orthopedic Disorders

Orthopedical disorders cause local pathological changes involving local factors in many cases. After significant damage has been caused to a bone structure, for example, during a major hip surgery, the resulting heterotropic ossification is likely caused by prostaglandin and can be reduced by inhibitors of prostaglandin [93]. Local prostaglandin and cytokine production by inflammatory cells can also be the cause of prosthesis loosening [94].

#### 2.7.5. Paget Disease

Paget disease expresses itself by an abnormal activation of osteoclasts, likely caused by viral infection [95]. This results in an irregular resorption pattern followed by an increased osteoblastic response to compensate for the heightened osteoclastic activity by an overproduction of woven bone. This may lead to an increase in bone density, but because of the irregular structuring, weaker zones and clusters may form, which may result in pathological fractures. There is also a genetic component to Paget disease linked to an osteosarcoma tumor suppressor gene [96], increasing the osteosarcoma risk for Paget disease patients.

## 3. Conclusions

Understanding the intricate interplay of structural, molecular, and functional aspects of bone is crucial for gaining a comprehensive knowledge of this tissue. In the course of this article, we have described the properties of bone tissue with emphasis on bone microscopy. This review can serve to familiarize the reader with the basics of anatomy, histology, and physiology of bone tissue and can help to understand the more complex aspects of this connective tissue.
